# Supplementary material for: Pupal behavior emerges from unstructured muscle activity in response to neuromodulation in Drosophila
Source: eLife. 2021 Jul 8;10:e68656. doi: 10.7554/eLife.68656 (PMC8331185; doi:10.7554/eLife.68656)
Supplement: Supplementary file 1. [file elife-68656-supp1.docx]

**Supplementary File 1: Pupal Neuromuscular Anatomy**

| Spatial  Group (N=17) | Muscle/  MN-IB (N=17) | CCAP-R+  MN (N=11) | Nerve | Segments (N=17) | CCAP-R+  Muscle (N=5) |
| --- | --- | --- | --- | --- | --- |
| D/DL | 1 | + | ISN^DM^ | A1-A7 | - |
| D/DL | 2 | + | ISN^DM^ | A1-A7 | - |
| D/DL | 3 | + | ISN^DM^ | A1-A7 | - |
| D/DL | 9 | + | ISN^DM^ | A1-A7 | - |
| D/DL | 10 | + | ISN^DM^ | A1-A7 | - |
| D/DL | 4* | - | ISN^DM^ | A1-A3 | - |
| V/VL | 12* | - | ISNb | A1-A4 | - |
| V/VL | 13 | + | ISNb | A1-A7 | - |
| V/VO | 28 | + | ISNb | A1-A7 | - |
| V/VO | 15 | + | ISNd | A1-A7 | - |
| L/DO | 5* | - | SNa | A1-A4 | - |
| L/TR | 8 | - | SNa | A1-A7 | - |
| L/TR | 21/22 | - | SNa | A1-A7 | + |
| L/TR | 22/23 | - | SNa | A1-A7 | + |
| L/TR | 23/24 | - | SNa | A1-A7 | + |
| V/TR | 25 | - | TN | A1-A7 | - |
| V/VA | 26 | - | SNc | A1-A7 | - |

a. Abbreviations: D, dorsal; L, lateral; V, ventral; DL, dorsal longitudinal; VL, ventral longitudinal; VO, ventral oblique; DO, dorsal oblique; TR, transverse; VA, ventral acute

b. * Degrades prior to pupal ecdysis in segments posterior to HS3 or HS4

c. N is the number of animals
