# Supplementary material for: Pupal behavior emerges from unstructured muscle activity in response to neuromodulation in Drosophila
Source: eLife. 2021 Jul 8;10:e68656. doi: 10.7554/eLife.68656 (PMC8331185; doi:10.7554/eLife.68656)
Supplement: Supplementary file 2. [file elife-68656-supp2.docx]

**Supplementary File 2: Variability of Phase Parameters**

| **Phase** | **Mean ± SD** | **Mean CV ± SD** | **N** |
| --- | --- | --- | --- |
| **P0** |  |  |  |
| Phase Duration (min) | - | - | 16 |
| Bout # | - | - | 10 |
| Bout Duration (sec) | 34.42 ± 9.45 | 0.41 ± 0.15 | 10 |
| IBI Duration (sec) | 8.25 ± 3.33 | 0.82 ± 0.33 | 10 |
| **P1** |  |  |  |
| Phase Duration (min) | 12.63 ± 3.45 | 0.27 | 16 |
| Bout # | 25.1 ± 5.17 | 0.51 ± 0.093 | 10 |
| Bout Duration (sec) | 31.43 ± 4.63 | 0.36 ± 0.061 | 10 |
| IBI Duration (sec) | 3.43 ± 1.32 | 0.72 ± 0.14 | 10 |
| **P2** |  |  |  |
| Phase Duration (min) | 5.68 ± 1.02 | 0.18 | 16 |
| Bout # | 9.1 ± 0.88 | 0.51 ± 0.099 | 10 |
| Bout Duration (sec) | 31.23 ± 4.01 | 0.32 ± 0.066 | 10 |
| IBI Duration (sec) | 5.33 ± 1.67 | 0.63 ± 0.12 | 10 |
| **P3** |  |  |  |
| Phase Duration (min) | - | - | 16 |
| Bout # | - | - | 10 |
| Bout Duration (sec) | 85.21 ± 16.32 | 0.38 ± 0.058 | 10 |
| IBI Duration (sec) | 6.89 ± 1.87 | 0.62 ± 0.1 | 10 |

a. Abbreviations: SD, standard deviation; CV, coefficient of variation

b. N is the number of animals
